# On the similarity between aortic and carotid pressure diastolic decay: a mathematical modelling study

**DOI:** 10.1038/s41598-023-37622-y

**Published:** 2023-07-04

**Authors:** Vasiliki Bikia, Georgios Rovas, Sokratis Anagnostopoulos, Nikolaos Stergiopulos

**Affiliations:** Laboratory of Hemodynamics and Cardiovascular Technology, Institute of Bioengineering, Swiss Federal Institute of Technology, Lausanne, Switzerland

**Keywords:** Biomarkers, Biomedical engineering, Computational science

## Abstract

Aortic diastolic pressure decay (DPD) has been shown to have considerable pathophysiological relevance in the assessment of vascular health, as it is significantly affected by arterial stiffening. Nonetheless, the aortic pressure waveform is rarely available and hence the utility of the aortic DPD is limited. On the other hand, carotid blood pressure is often used as a surrogate of central (aortic) blood pressure in cardiovascular monitoring. Although the two waveforms are inherently different, it is unknown whether the aortic DPD shares a common pattern with the carotid DPD. In this study, we compared the DPD time constant of the aorta (aortic RC) and the DPD time constant of the carotid artery (carotid RC) using an in-silico-generated healthy population from a previously validated one-dimensional numerical model of the arterial tree. Our results demonstrated that there is near-absolute agreement between the aortic RC and the carotid RC. In particular, a correlation of ~ 1 was reported for a distribution of aortic/carotid RC values equal to 1.76 ± 0.94 s/1.74 ± 0.87 s. To the best of our knowledge, this is the first study to compare the DPD of the aortic and the carotid pressure waveform. The findings indicate a strong correlation between carotid DPD and aortic DPD, supported by the examination of curve shape and the diastolic decay time constant across a wide range of simulated cardiovascular conditions. Additional investigation is required to validate these results in human subjects and assess their applicability in vivo.

## Introduction

During diastole, the blood flow at the level of the proximal aorta comes to a halt, resulting in an exponential decay of arterial pressure. This decay is characterized by a time constant, which is determined by the product of peripheral resistance, R, and the total arterial compliance, C, namely *τ* = RC. As the arterial pressure pulse travels through the systemic circulation, it undergoes significant variations, with the diastolic pressure decay (DPD) often exhibiting a non-monotonic behavior over time^1,2^. However, a relatively smooth DPD has been observed for the aortic pressure pulse^3^.

Aortic DPD has been shown to have considerable pathophysiological relevance, as it is significantly affected by arterial stiffening due to two main factors: (1) first, in normal conditions during left ventricular contraction, a great quantity of blood is stored within the aorta and the large elastic arteries until being released in order to maintain physiological pressure values during diastole (Windkessel effect)^4^, and (2) second, with arterial stiffening the reflected waves return earlier in the ascending aorta and largely overlap with the forward wave during the systolic phase, leading to a further rapid decay of pressure in diastole^5^.

A large majority of methods to estimate arterial compliance are based on the assumption that the vasculature behaves like a Windkessel model in which the aortic diastolic pressure decays exponentially with the DPD time constant RC^6–8^. As a result, a strong link between arterial compliance and diastolic decay of the aortic pressure waveform has been established. This relationship has inspired various methods to monitor vascular health, including the monitoring of central hemodynamics, such as cardiac output. For instance, Bourgeois et al. introduced a study in which the aortic DPD is utilized for achieving the continuous monitoring of changes in peripheral vascular resistance^9^, and thus cardiac output.

Nonetheless, the aortic pressure waveform is rarely available and hence the utility of aortic DPD is limited. Carotid blood pressure is often used as a surrogate of central (aortic) blood pressure when direct measurement of aortic pressure is challenging or impractical. Especially, it serves as a convenient alternative when continuous monitoring of aortic hemodynamics is required, such as during surgeries or in critical care. Additionally, carotid pressure can be used as a substitute in research studies and clinical trials where direct aortic pressure measurements are not feasible. While the carotid and aortic waveforms differ in location, waveform characteristics, and reflectance properties, it remains uncertain if the aortic DPD follows a similar pattern to the carotid DPD. The carotid waveform is impacted by local factors, while the aortic waveform reflects systemic circulation effects. Notably, as the pressure wave travels through the arterial system, encountering branch points and impedance changes, reflections arise, resulting in modifications to the shape and characteristics of the aortic waveform. In the present study, we compared the DPD of the aorta (aortic RC) and the one of the common carotid artery (carotid RC). To the best of our knowledge, there are no previously published data comparing the above, which is mainly attributed to the intrinsic difficulty in acquiring concurrent invasive pressure data within the aorta and at the carotid level. In silico studies offer a cost-effective and efficient means to explore novel concepts and hypotheses across a wide range of cardiovascular parameters. Considering the cost and complexity associated with simultaneous measurements of aortic and carotid blood pressure in a clinical setting, this study overcomes this limitation by using in silico data from a previously validated cardiovascular computer simulator^10^.

## Methods

### In silico data

This study utilized a synthetic dataset which was previously designed and generated to simulate various hemodynamical states^11^. A diverse range of hemodynamic scenarios corresponding to 3818 virtual individuals, encompassing both normotensive and hypertensive adults, were simulated. This was achieved by modifying key cardiac and systemic parameters within a previously validated in silico cardiovascular model^10^. The model's parameters were varied by mining literature data and applying random Gaussian sampling to introduce variation. Table 1 displays the input parameters of the one-dimensional (1-D) cardiovascular model, along with the chosen range of variation for each parameter. Cardiac parameters were altered and different cardiac output values were simulated. Arterial geometry (i.e. arterial length and diameter) was modified to represent variations in arterial tree sizes and body types^12,13^. Total peripheral resistance and arterial distensibility were altered based on relevant literature sources^14–16^. Especially, variation of arterial distensibility of all arteries was performed in a uniform manner with respect to the variation of the aortic distensibility. In specific cases, the model accounted for non-uniform and more pronounced aortic stiffening, as previously described in related works^17,18^, to simulate older or hypertensive individuals. By inputting a specific set of parameters, the model generated analytical solutions for pressure and flow at each arterial segment. The physiological validity of each simulated subject was evaluated by comparing the simulated brachial and aortic systolic blood pressure (SBP), diastolic blood pressure (DBP), mean arterial pressure (MAP), and pulse pressure (PP) against reference values reported in prior studies by McEniery^19^ (normotensive cases) and Bordin Pelazza and Filho^20^ (hypertensive cases). To ensure a comprehensive analysis, we intentionally included a wide range of parameters, encompassing both typical and extreme scenarios. Therefore, any subject whose blood pressure values fell outside the 99.5% confidence intervals (mean ± 2.807SD) was excluded from the dataset. To generate the dataset, the model was executed 10000 times, resulting in the creation of 10000 cases. After applying the aforementioned filtering criteria, a total of 3818 samples were deemed acceptable out of the initial 10000 cases.

**Table 1:** List of input parameters of the 1-D cardiovascular model.

Input model parameter	Selected range	References
End-systolic elastance (mmHg/mL)	[1.03, 3.50]	^21–23^
End-diastolic elastance (mmHg/mL)	[0.05, 0.20]	
Filling pressure (mmHg)	[7, 23]	^24^
Time of maximal elastance (ms)	340	^25^
Heart rate (bpm)	[60, 100]	–
Dead volume (mL)	15	^24^
Venous resistance (mmHg s/mL)	0.003	^10^
Total arterial compliance (mL/mmHg)	[0.10, 3.80]	^15,16^
Total peripheral resistance (mmHg s/mL)	[0.5, 2]	^14^
Arterial inlet diameter (cm)^a^	[1.9, 4]	^12,13^
Arterial outlet diameter (cm)^a^		
Arterial length (cm)^b^	[150, 200]	–
Blood density (kg/m^3^)	1050	–
Blood viscosity (Pa s)	0.004	–

^a^Arterial diameter was altered with respect to the diameter of the aorta. The alteration of the diameter across all arteries was done uniformly.

^b^Arterial length was modified with respect to the height. The reference state of the arterial tree model corresponds to an individual with a height equal to 180 cm.

Blood pressure waveforms were obtained from the virtual aortic root and the virtual left common carotid artery. From the simulated pulse at each artery, the SBP, DBP, and PP were extracted for both the aorta and the left common carotid artery. The total peripheral resistance was derived as the ratio of MAP over cardiac output (CO). The total arterial compliance (C) was computed analytically as the sum of volume compliance (c_i_) of all the arterial segments included in the 1-D mathematical model and the terminal compliances described by the terminal Windkessel models^4^.

### Derivation of the RC

**Figure 1:**
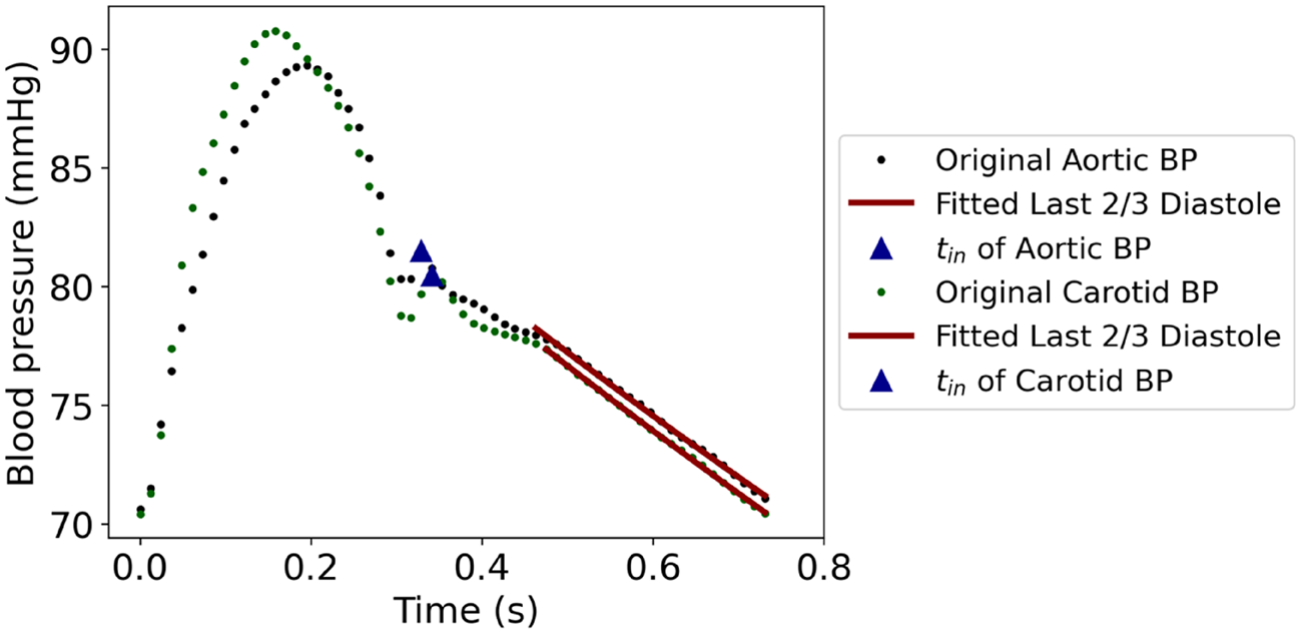
Fitting a mono-exponential decay function to the last 2/3 of the diastole of the aortic and carotid blood pressure (BP) waveform.

The DPD time constant, RC, was calculated from the blood pressure waveform at the two arterial sites (aortic root and common left carotid artery) by fitting a mono-exponential decay function to the last 2/3 of the diastole. The choice of the last part of the diastole was based on the findings of a previous study of Stergiopulos et al.^6^. In particular, the authors found that the later part of the diastole, namely from the time that the systolic pressure wave has reached all peripheral beds, provides the most precise results. Based on the two-element Windkessel principle, the earliest starting time for the RC estimation can be the time when flow is zero, namely end of the incisura (t_in_). Therefore, the diastolic part of each pressure pulse was selected to be denoted by t_in_ (starting point) and the end of the cycle (ending point). The last 2/3 of the diastole was then isolated to perform the curve fitting. For the sake of completeness, we also assessed the RC derived using two additional selected parts of the curve, namely the last 1/3 of the diastole, and the entire part after t_in_ (Fig. 1).

Moreover, we wished to assess the agreement of the aortic RC and the RC estimated from a rescaled carotid blood pressure waveform. Precisely, the carotid blood pressure waves of the entire population were normalized between 0 and 1 (using the min–max formulation) and, subsequently, were scaled using the simulated cuff blood pressure, i.e. brachial SBP and DBP. The calibration was based on the assumption that DBP and MAP remain relatively constant across all major arteries. The MAP was calculated as (SBP + 2DBP)/3. Comparison between the aortic RC and the calibrated carotid RC was performed anew. All the aforementioned experiments were also performed under the assumption that the carotid blood pressure data were corrupted with artificial random Gaussian noise (μ = 0, σ = 0.3) s.

### Statistical analysis

Data are presented as mean and standard deviation (SD). The pairs of DPD time constants, namely RC values, were compared by using the mean absolute error (MAE), Pearson’s correlation coefficient (r), and the Bland–Altman analysis^26^. We performed linear least-squares regression for the aortic RC and the carotid RC data. The slope and the intercept of the regression line were reported. A two-sided p-value for a hypothesis test whose null hypothesis is that the slope is zero using Wald Test with t-distribution of the test statistic was also calculated. The level of statistical significance was set to be less than 0.05. The statistical analysis was implemented in Python (Python Software Foundation, Python Language Reference, version 3.6.8, Available at http://www.python.org).

## Results

**Figure 2:**
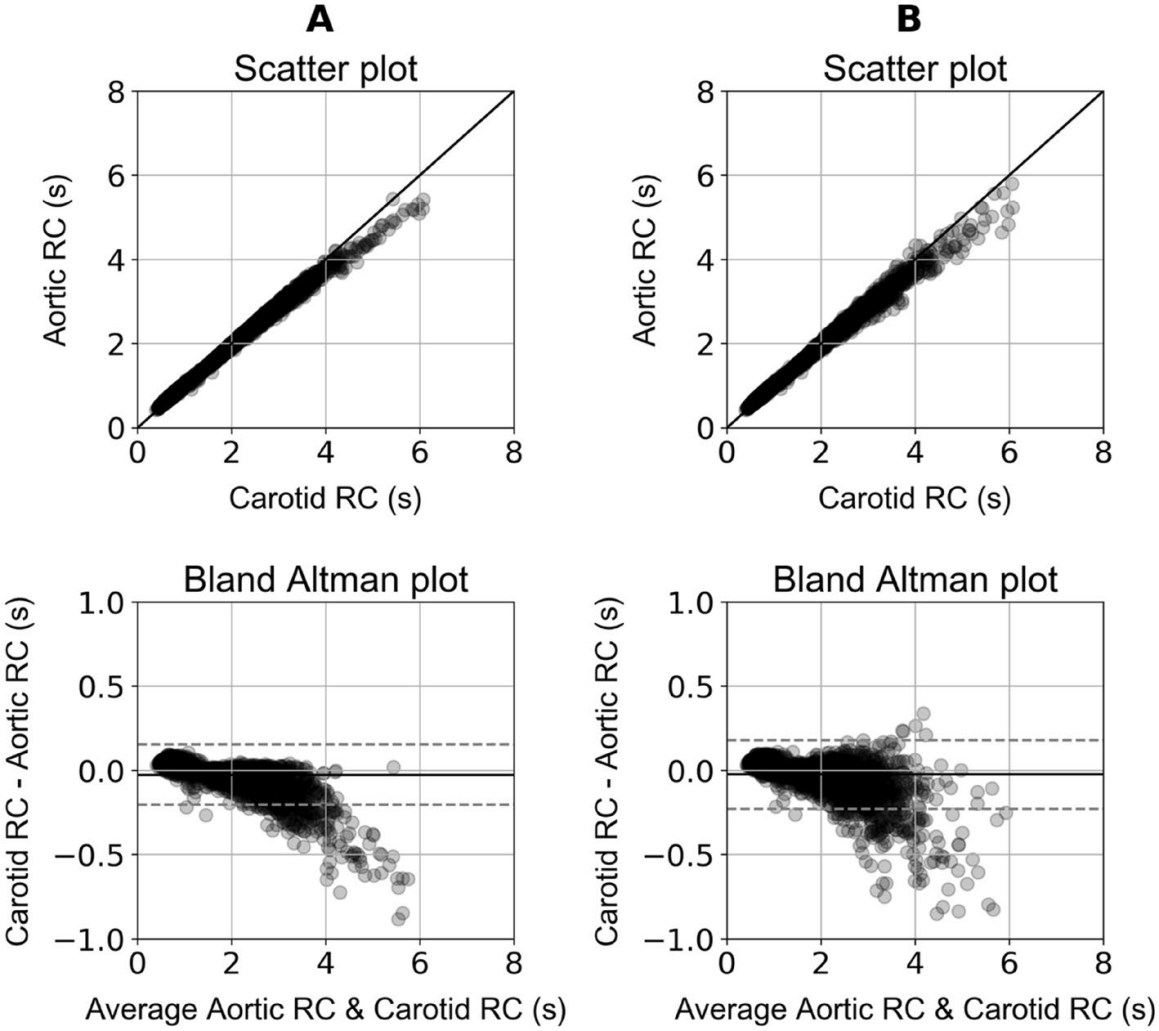
Scatter plots and Bland Altmans plot comparing the aortic RC values with: (**A**) Noise-free carotid RC [*2/3 diastole*], (**B**) Noisy carotid RC [*2/3 diastole*].

**Figure 3:**
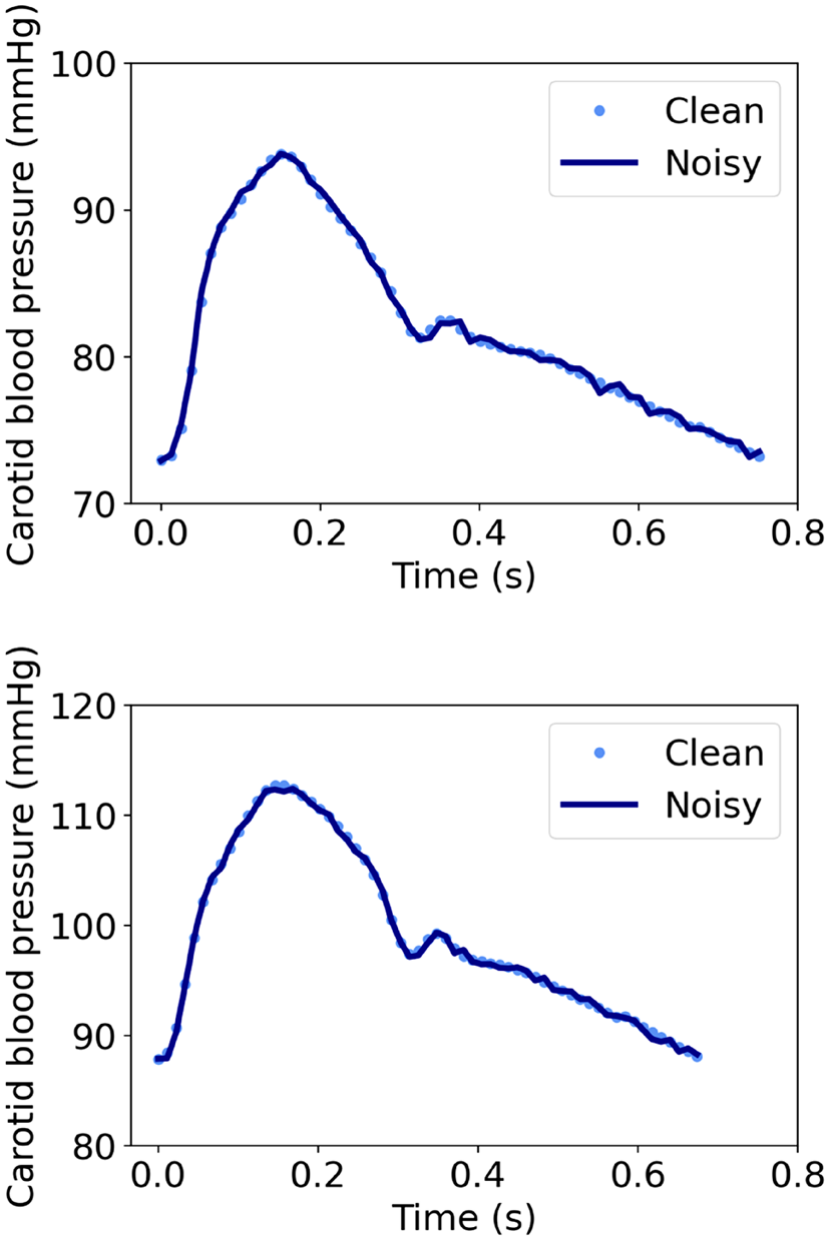
Carotid blood pressure waveforms distorted with artificial random Gaussian noise (μ = 0, σ = 0.3) s.

Table 2 summarizes the distributions of the cardiovascular parameters for the in silico population. Aortic RC values and carotid RC values (using the 2/3 of the diastole for the RC estimation) were reported to be 1.76 ± 0.94 s and 1.74 ± 0.87 s, respectively. Distributions of all RC values are presented in Table 2. The comparison between the aortic RC and the noise-free carotid RC values is presented in Fig. 2A. Correlation coefficient was found to be ~ 1 (absolute agreement). For the noise-free data, the slope and the intercept of the regression line were equal to 0.92 (*p* < 0.0001) and 0.11 s, respectively, while the Bland–Altman analysis indicated a bias of − 0.02 s and limits of agreement, within which 95% of errors are expected to lie, were found to be equal to ± 0.2 s. In addition, correlation between the aortic RC and the noisy carotid RC was reported to be close to 1, whereas the Bland–Altman analysis yielded a close-to-zero bias equal to − 0.02 s and narrow limits of agreement of [− 0.23, 0.18] s (Fig. 2B). Figure 3 presents two carotid blood pressure pulses which were corrupted with artificial random Gaussian noise. Accuracy, correlation, and bias metrics are aggregated in Table 3 for all the performed experiments. No significant variation in accuracy was reported for the different selected curve parts and all considered parts allowed the estimation of the DPD time constant across all simulated waveforms, while avoiding negative asymptotic values.

**Table 2:** Description of the cardiovascular characteristics of the study cohort (n = 3818).

Variable	Mean (μ)	Standard deviation (SD)
	n = 3818	
Aortic RC [*1/3 diastole*] (s)	1.73	0.83
Aortic RC [*2/3 diastole*] (s)	1.76	0.94
Aortic RC [*entire diastole*] (s)	1.89	1.08
Carotid RC [*1/3 diastole*] (s)	1.72	0.79
Carotid RC [*2/3 diastole*] (s)	1.74	0.87
Carotid RC [*entire diastole*] (s)	1.86	1.03
Calibrated carotid RC [*1/3 diastole*] (s)	1.76	0.74
Calibrated carotid RC [2*/3 diastole*] (s)	1.77	0.83
Calibrated carotid RC [entire *diastole*] (s)	1.89	0.99
Aortic systolic blood pressure (mmHg)	123	24
Aortic diastolic blood pressure (mmHg)	81	21
Aortic pulse pressure (mmHg)	42	19
Carotid systolic blood pressure (mmHg)	124	23
Carotid diastolic blood pressure (mmHg)	80	21
Carotid pulse pressure (mmHg)	45	19
Brachial systolic blood pressure (mmHg)	135	24
Brachial diastolic blood pressure (mmHg)	77	21
Brachial pulse pressure (mmHg)	57	23
Aortic mean arterial pressure (mmHg)	101	21
Heart rate (bpm)	73	15
Total arterial compliance (mL/mmHg)	1.14	0.47
Total peripheral resistance (mmHg s/mL)	1	0.21
Stroke volume (mL)	81	8
Aortic characteristic impedance (mmHg.s/mL)	0.056	0.012
Local aortic pulse wave velocity (m/s)	5.15	1.36
Local carotid pulse wave velocity (m/s)	7.06	1.86

**Table 3:** Accuracy, correlation, and bias metrics.

Aortic DPD versus	r	MAE (s)	Slope (*p-*value)	Intercept (s)	Bias (LoA) (s)
*Noise-free carotid pressure data*					
Carotid DPD [*1/3 diastole*]	1	0.04	0.95 (*p* < 0.0001)	0.07	− 0.01 [− 0.14, 0.12]
Carotid DPD [*2/3 diastole*]	1	0.05	0.92 (*p* < 0.0001)	0.11	− 0.02 [− 0.2, 0.15]
Carotid DPD [*entire diastole*]	1	0.06	0.95 (*p* < 0.0001)	0.06	− 0.03 [− 0.18, 0.12]
Calibrated carotid DPD [*1/3 diastole*]	0.99	0.11	0.88 (*p* < 0.0001)	0.22	0.02 [− 0.27, 0.31]
Calibrated carotid DPD [2*/3 diastole*]	0.99	0.11	0.87 (*p* < 0.0001)	0.23	0.01 [− 0.32, 0.33]
Calibrated carotid DPD [entire *diastole*]	0.99	0.11	0.91 (*p* < 0.0001)	0.16	− 0.0 [− 0.29, 0.29]
*Noisy carotid pressure data*					
Carotid DPD [*1/3 diastole*]	0.98	0.09	0.96 (*p* < 0.0001)	0.07	− 0.01 [− 0.29, 0.28]
Carotid DPD [*2/3 diastole*]	1	0.06	0.93 (*p* < 0.0001)	0.11	− 0.02 [− 0.23, 0.18]
Carotid DPD [*entire diastole*]	1	0.06	0.96 (*p* < 0.0001)	0.05	− 0.03 [− 0.2, 0.14]
Calibrated carotid DPD [*1/3 diastole*]	0.98	0.14	0.91 (*p* < 0.0001)	0.2	0.04 [− 0.31, 0.4]
Calibrated carotid DPD [2*/3 diastole*]	0.99	0.11	0.89 (*p* < 0.0001)	0.21	0.02 [− 0.29, 0.33]
Calibrated carotid DPD [entire *diastole*]	0.99	0.1	0.93 (*p* < 0.0001)	0.14	0.02 [− 0.27, 0.3]

r: Pearson's correlation coefficient; MAE: mean absolute error; LoA: limits of agreement.

**Figure 4:**
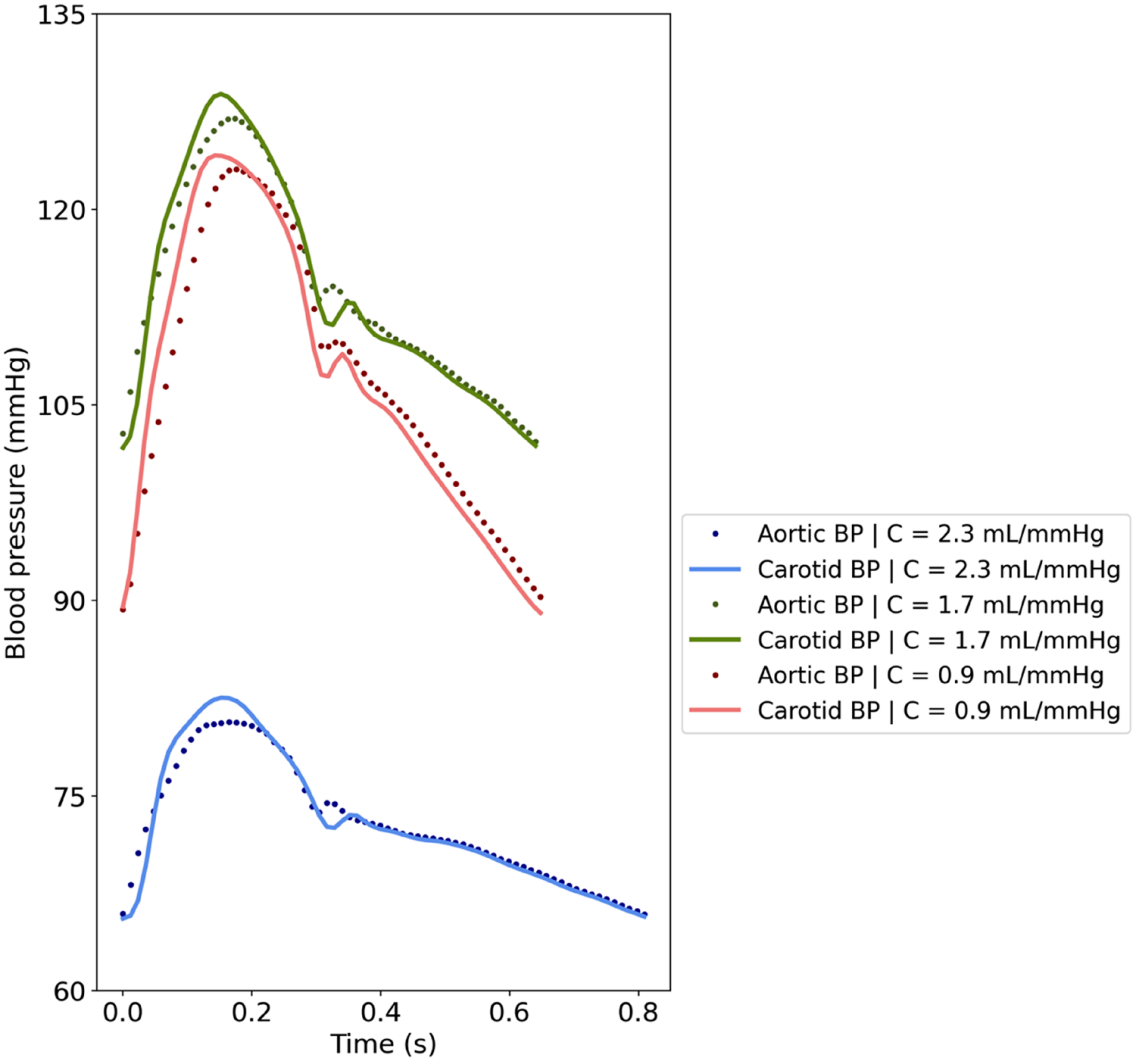
Comparison of aortic and carotid blood pressure (BP) waveforms for different values of total arterial compliance (C).

We additionally present indicative examples of simulated pressure waveforms to enable comparison of the shape of the two pressure pulses. Figure 4 directly compares the blood pressure waveforms at the two arterial sites, namely the aorta and the common left carotid artery, for three different levels of total arterial compliance (C), namely a highly compliant arterial tree with C = 2.3 mL/mmHg, a compliant arterial tree with C = 1.7 mL/mmHg, and a stiff arterial tree with C = 0.9 mL/mmHg.

## Discussion

This study demonstrated that there is excellent agreement between the carotid diastolic decay time constant and the aortic diastolic decay time constant in an in silico population of normotensive and hypertensive virtual subjects generated using a previously validated 1-D cardiovascular numerical model. A total of 3818 realistic virtual subjects, representing a wide variety of hemodynamical profiles, was assessed. The simulated data allowed for simultaneous acquisition of the aortic and carotid blood pressure signals, mitigating prevalent limitations that occur in real human data collection processes.

In the current study, we compared three scenarios, where the DPD time constant was calculated from three different selected diastolic parts. Across all scenarios, the agreement between the aortic and carotid RC values remained consistently high, highlighting the reliability and validity of the study’s results. The carotid RC values were found to be slightly lower in comparison to the aortic RC values for slower diastolic pressure decays. Furthermore, the addition of artificial random Gaussian noise led to the distortion of the diastolic part of the blood pressure waves. The scenario where the last 1/3 of the diastole was selected to derive the RC had the highest sensitivity to added noise. This is to be expected as using a smaller, noise-distorted portion for the RC estimation hampers the ability to assess properly the DPD pattern. In addition, it was of interest to investigate the sensitivity of the methodology’s precision when the carotid RC was derived from a rescaled carotid blood pressure waveform (using the simulated cuff-based pressure measurement). The deviation in the amplitude of the rescaled carotid blood pressure pulse did not essentially affect the accuracy in the RC estimation. Although there was slightly less agreement compared to the results obtained using the original waveform, the overall agreement remained high.

The inherent limitations in obtaining an accurate recording of the aortic blood pressure discourage the utility of the aortic DPD in the clinical practice. As arterial blood pressure pulse varies essentially during its transmission throughout the systemic circulation, investigation of a possible similarity between the diastolic parts of pulse waves at different arterial sites would be of high interest. In a previous study^27^, Izzo et al. compared the radial and the carotid diastolic decay values in 75 subjects to determine if the diastolic decay time constant is systemic or site-specific. Their results indicated that the two values are not correlated, although they are both affected by arterial stiffening. These findings suggested that local factors substantially affect DPD properties and that a systemic diastolic decay time constant cannot be determined from a local peripheral pressure waveform without further modification. However, no previous work has compared the DPD in simultaneous recordings of aortic and carotid blood pressure waveforms. Accessing the information of aortic DPD from a more accessible and non-invasive pressure measurement may introduce new possibilities in the utility of the RC time constant to evaluate vascular age biomarkers and potentially enable new patient stratification approaches under different clinical scenarios.

Evaluation of the hemodynamical function of the cardiovascular system via measurement of the mechanical properties of the large arteries (such as the aortic RC) may provide a substantial improvement over existing monitoring techniques. Future research directions may involve leveraging the findings of this study in conjunction with modern sensors^28,29^. These sensors could encompass wearable devices such as necklaces or collars, employing diverse technologies (e.g. ultrasound patches and optical sensors) to enable non-invasive recording of the carotid blood pressure waveform. Subsequently, the recorded carotid blood pressure pulse may enable the assessment of central pulse waveform parameters that are expected to replace approximated central pulse waves derived from peripheral blood pressure measurement using the inflatable cuff (based, for instance, on generalized transfer functions). Notably, cuff-based data, such as measurements taken at the brachial artery using oscillometric or sphygmomanometric methods, do not serve as desirable indicators, as there are currently no available data supporting a direct relationship between aortic RC and RC derived from peripheral blood pressure signals. Albeit cuff-enabled devices have the capability to provide indirect measurements of aortic blood pressure, and consequently, aortic RC, the feasibility of non-invasive and easier access to the carotid artery renders the use of the proposed application more advantageous. By focusing on the carotid artery, which offers a simpler and more accessible site for measurement, the process can be streamlined, potentially enhancing the accuracy of assessing aortic hemodynamics.

Carotid blood pressure is considered as a well-established surrogate of central blood pressure and it is frequently used as a replacement to the aortic blood pressure measurement. In a previous study, common carotid arterial stiffness, as assessed by carotid ultrasonography, has been shown to be fairly correlated with aortic stiffness^30^. This evidence strengthens the clinical importance of carotid stiffness in cardiovascular risk assessment, by supporting its validity as a surrogate for aortic stiffness and by offering an alternative strategy to estimate aortic stiffness in the clinic. Motivated by the aforementioned, the present study adds to the literature by providing a complete comparison between the aortic and carotid DPD across a wide range of hemodynamical profiles. We demonstrated that the diastolic part of the carotid blood pressure wave overlaps with the diastolic part of the aortic blood pressure wave, despite reported differences in other waveform characteristics, especially in the early systole (Fig. 4). Prominent differences between the two waveforms were noted in characteristics such as the pressure peaks, timing of pressure peaks’ occurrence, and the upstroke in the early systole.

From a clinical perspective, diastolic decay time has been previously shown to bear essential pathophysiological information. For instance, aortic stiffening with reduced compliance is likely to cause damage to myocardial function by accelerating the diastolic decay of the central blood pressure; thus increasing the risk of ischemic heart disease in hypertensive patients^31^. Moreover, diastolic time is a major factor to be considered in the regulation of the myocardial flow supply–demand ratio, as blood flow beneath the endocardium occurs nearly entirely during the diastolic phase. Providing a viable alternative to the aortic diastolic decay information, such as using easily obtained non-invasive recordings in the ambulatory care (e.g. tonometric measurement of the carotid blood pressure signal), may enable new opportunities to assess vascular health.

Replacement of the aortic diastolic decay time by the carotid diastolic decay time constant could also facilitate and improve existing methods for estimating total arterial compliance. In particular, a considerable number of compliance monitoring techniques relies on the derivation of the aortic DPD. These approaches suffer from two inherent challenges: (i) acquiring the aortic blood pressure waveform, which is done either invasively or is approximated using a mathematical transformation of a peripheral (radial or brachial) blood pressure waveform, and (ii) obtaining recordings of the aortic blood flow—simultaneously with aortic blood pressure recordings—in order to estimate total peripheral resistance. On the other hand, tonometry is turning into a popular technique for blood pressure monitoring and could provide a relatively easy, repeatable, non-invasive alternative to derive a surrogate of central DPD.

The main limitation of the present study pertains to the use of in silico data to perform the analysis. Translation and application of the theoretical results from any in silico study to clinical conditions should not be direct. Yet, in silico models possess several advantages; e.g. they provide high-quality, noise-free signals; they allow for controlling specific variables in highly multifactorial problems; and they give access to simulated measurements which are difficult to obtain under in vivo conditions. In this study, the in silico cardiovascular model permitted the generation of a virtual population covering an extensive variety of realistic cardiovascular conditions. Importantly, the mathematical model that was used to generate the specific in silico population analyzed in this study has been thoroughly validated against in vivo data and provides realistic representations of the physiological blood pressure and flow signals.

## Conclusion

The present study demonstrates that the carotid DPD shows excellent agreement with the aortic DPD, substantiated through comparison of the curve shape and the diastolic decay time constant, RC, across diverse simulated cardiovascular conditions. This is an inaugural attempt to directly compare the diastolic decay of the aortic and the carotid pressure waveforms. Further evaluation of our findings remains to be conducted in humans to verify their validity in vivo.

## Data Availability

The dataset used and analysed in the current study is available from the corresponding author on reasonable request.
